# iPod-based in-home system for monitoring gaze-stabilization exercise compliance of individuals with vestibular hypofunction

**DOI:** 10.1186/1743-0003-11-69

**Published:** 2014-04-21

**Authors:** Kevin Huang, Patrick J Sparto, Sara Kiesler, Daniel P Siewiorek, Asim Smailagic

**Affiliations:** 1 Carnegie Mellon University, 5000 Forbes Ave, Pittsburgh, PA 15213, USA; 2 University of Pittsburgh Medical Center, 200 Lothrop St, Pittsburgh, PA 15213, USA

**Keywords:** iPod, iPhone, Mobile computing, Vestibular rehabilitation, Gaze-stabilization exercise, Balance, Dizziness, Monitoring

## Abstract

**Background:**

In the physical therapy setting, physical therapists (PTs) often prescribe exercises for their clients to perform at home. However, it is difficult for PTs to obtain information about their clients’ compliance with the prescribed exercises, the quality of performance and symptom magnitude. We present an iPod-based system for capturing this information from individuals with vestibular hypofunction while they perform gaze stabilization exercises at home.

**Method:**

The system’s accuracy for measurement of rotational velocity against an independent motion tracker was validated. Then a seven day in-home trial was conducted with 10 individuals to assess the feasibility of implementing the system. Compliance was measured by comparing the recorded frequency and duration of the exercises with the exercise prescription. The velocity and range of motion of head movements was recorded in the pitch and yaw planes. The system also recorded dizziness severity before and after each exercise was performed. Each patient was interviewed briefly after the trial to ascertain ease of use. In addition, an interview was performed with PTs in order to assess how the information would be utilized.

**Results:**

The correlation of the velocity measurements between the iPod-based system and the motion tracker was 0.99. Half of the subjects were under-compliant with the prescribed exercises. The average head velocity during performance was 140 deg/s in the yaw plane and 101 deg/s in the pitch plane.

**Conclusions:**

The iPod-based system was able to be used in-home. Interviews with PTs suggest that the quantitative data from the system will be valuable for assisting PTs in understanding exercise performance of patients, documenting progress, making treatment decisions, and communicating patient status to other PTs.

## Background

Complaints of dizziness and vertigo are common in the general population, with yearly prevalence rates of up to 25% [1,2]. People with vestibular disorders are more likely to experience dizziness and to fall than people without vestibular disorders [3,4]. Individualized vestibular-rehabilitation exercise programs are the standard of care for rehabilitation of persons with dizziness [5-7]. Often, a physical therapist (PT) will prescribe gaze-stabilization exercises for the patient to perform at home. Gaze-stabilization exercises involve moving the head horizontally or vertically (i.e., in the yaw and pitch planes) in a sinusoidal pattern while maintaining a fixed gaze on a visual target. The purpose of these exercises is to adapt an impaired vestibulo-ocular reflex (VOR) gain, restore the symmetry of dynamic vestibulo-ocular balance, and/or habituate the patient to motion-induced symptoms [8]. The prescription may include the direction of movement (yaw, pitch), the duration of movement (30 to 60 seconds), and the number of repetitions (several up to tens of repetitions per day) [9]. Movement characteristics such as range of motion, velocity, and frequency of movement may also be prescribed. As the patient recovers, the prescription can be progressed by increasing the velocity or frequency of movement and the duration or number of repetitions. Performing the exercises at home is considered to be an essential part of the rehabilitation program. The accumulated daily repetitions constitute a much greater dose than what can be done in the clinic once or twice a week, and are therefore a much greater stimulus for recovery.

When patients return for weekly or bi-weekly follow-up treatment visits, the physical therapist reassesses the patients’ status by inquiring about their symptom severity and interviewing them as to their compliance with the prescription. Metrics such as daily symptom severity and self-reported length and duration of exercise performance can be recorded using a daily-written exercise diary. However, direct measurements of home exercise behavior, such as the velocity, range of motion, and frequency of head movements, has not been obtainable. Measurements of prescription compliance and symptom severity are important for the therapist to know because they influence both long-term planning and the prescription that will be given for the upcoming week.

Passive activity monitoring offers a potentially simple solution for assessing patients’ compliance with their home exercise prescriptions. Activity monitoring today, however, often relies on camera-based systems, including video and motion cameras [10,11] or lab-prototyped custom sensors [12-15]. In the area of camera-based activity detection, Cucciara and colleagues explored techniques for the automatic video extraction of moving objects and people [10], and Goffredo and colleagues explored techniques for evaluating balance strategies and postural sway [11]. However, camera-based systems can be error-prone when providing fine measurements of rotation and acceleration. For example, the Microsoft Kinect SDK, an increasingly popular physical therapy research platform [16-18], currently does not offer head-rotation tracking due to camera-based limitations [19]. Camera-based systems can also be difficult for technologically inexperienced patients to use, as these systems usually must be connected and configured with a computer or other hardware. Usability is especially problematic for older adults, who want technology to be as simple and streamlined as possible [20]. Consequently, although there are many studies exploring the use of video and motion camera systems to measure exercise performance, including those using the Microsoft Kinect and the Nintendo Wii [21], these studies are mainly in-lab experiments rather than home deployments.

Another approach to activity monitoring uses custom sensor-based devices that can be worn or carried and are therefore more mobile than camera-based systems. Sensors on these devices may include accelerometers, gyroscopes, magnetometers and electro-active textiles. Researchers have examined a wide array of wearable technology for rehabilitation exercise monitoring, including electro-active garments and sensor networks [22,23]. Specifically, in the field of accelerometer-based applications, researchers have explored the use of multi-axial accelerometers for classifying basic movements, including walking, sitting, standing and falls [24,25]. However, similar to the camera-based system studies mentioned previously, research with such devices has mainly examined in-lab use rather than their feasibility for deployment in homes. These devices are also rarely self-contained and often must be connected to and configured with other hardware.

In this paper, we present an alternative in the form of a simple iPod-based sensor system. The iPod Touch 4G (101 grams, less than half the weight of a roll of quarters, which weighs 227 grams) is fitted into a baseball cap and worn on the head. In this first application, the system has been designed to monitor gaze-stabilization exercises in the home. The advantages of this approach include:

1. The system is self-contained to maximize simplicity. The interface (audio, video, touch) and sensors (accelerometer, gyroscope) are packaged together in the iPod, minimizing configuration complexity and increasing ease of use.

2. All relevant measures are integrated and recorded using the same application.

3. The system speeds development and deployment. By leveraging a common platform (iOS) and device (iPod), this approach lowers the barrier to development and real-world adoption.

In this paper, we describe the research testing the feasibility of using this commercial off-the-shelf (COTS) product for in-home gaze-stabilization exercise monitoring. We validated the iPod’s measurement of head velocity in the yaw and pitch planes by comparison with an externally validated sensor. We then performed a usability trial in which ten individuals with vestibular dysfunction used the system at home for a week, and we monitored their exercise compliance and performance. Finally, we interviewed physical therapists and assessed their feedback on data gathered by the system.

## Methods

### System architecture

**Figure 1:**
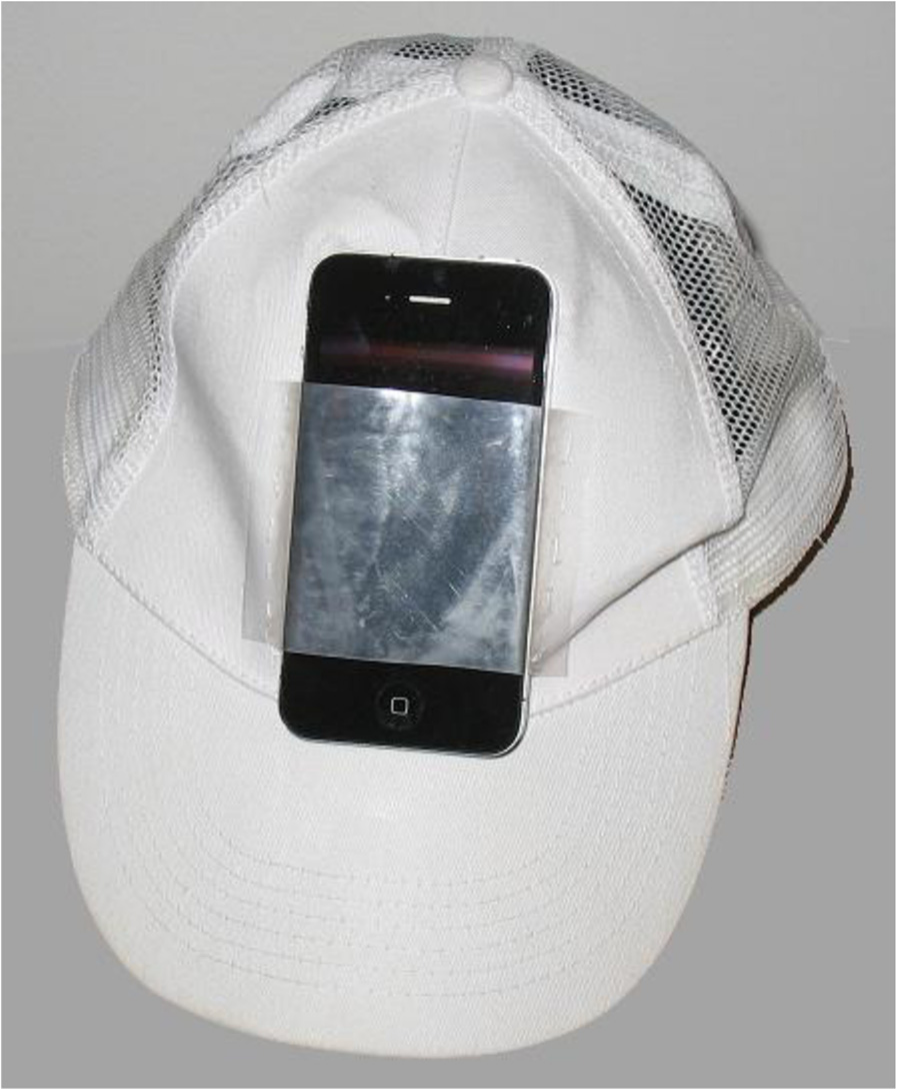
The system design, showing iPod placed in sleeve attached to front of cap.

**Figure 2:**
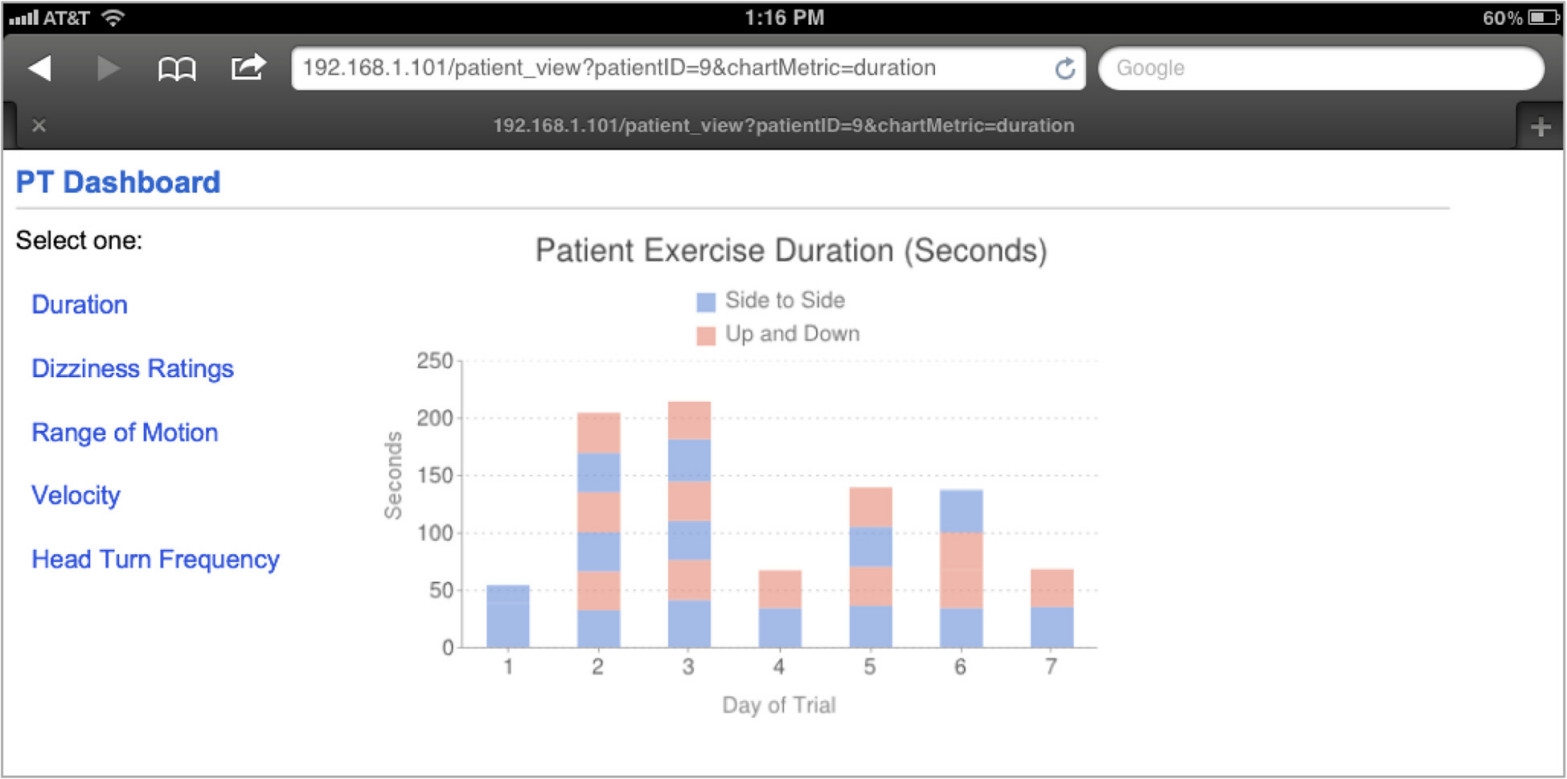
The PT Dashboard on the iPad, showing patient exercise statistics.

The prototype, as depicted in Figure 1, has three components: an iPod Touch 4G, a cap with a sewn-in sleeve to hold the iPod, and a custom software application. Patients wear the cap while they practice their exercises. They can operate the iPod through the clear plastic sleeve. When patients return to the clinic, data from the iPod are transferred to a centralized server via Wi-Fi and visualized on an iPad Dashboard so that the supervising physical therapist can review the exercise data with their patients. This PT Dashboard on the iPad is shown in Figure 2.

### iPod application implementation

**Figure 3:**
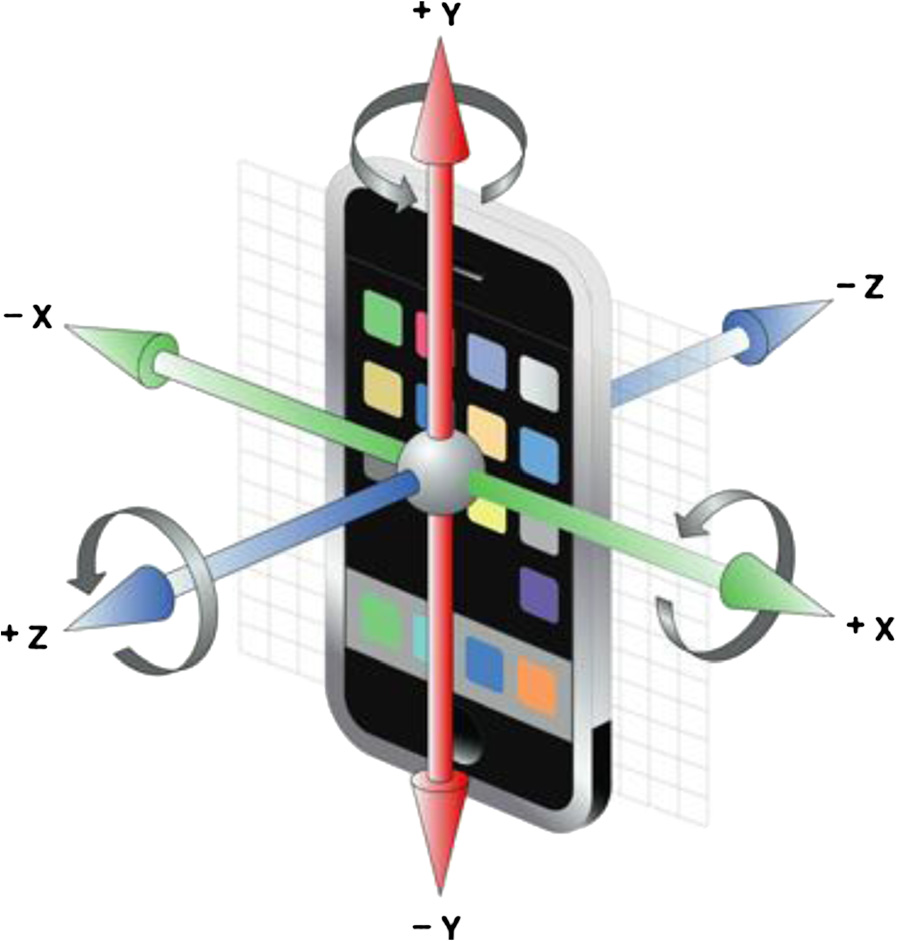
iPod rotation axes.

The iPod Touch 4G contains a tri-axial accelerometer and a tri-axial gyroscope. The iOS 4 SDK provides sensor-fused rotation-rate readings through the *rotationRate* property of the CMDeviceMotion object. This property combines both accelerometer and gyroscope data via Apple’s sensor-fusion algorithm to provide a more accurate rotation rate than can be acquired from the gyroscope alone. The rotation rates are about the iPod’s reference frame, shown in Figure 3. We configured the software to sample this *rotationRate* at 60 hz, which was achieved by the application during run-time with small fluctuations.

**Figure 4:**
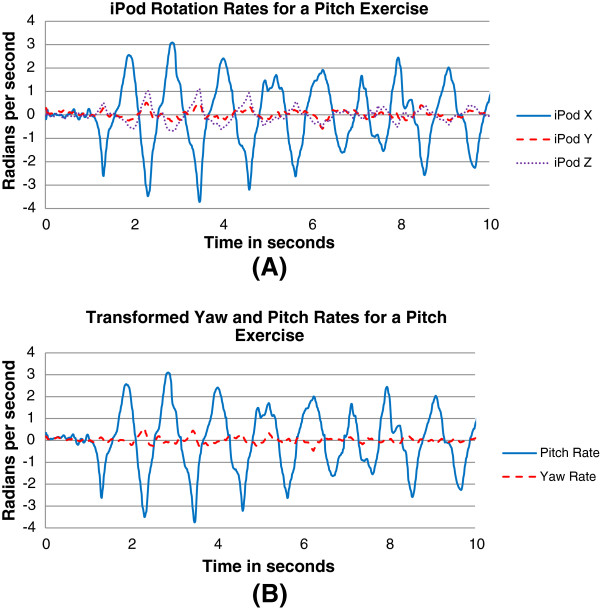
**Sample traces showing rotation rate values of the gaze-stabilization exercise for pitch movements. (A)** iPod X, Y, and Z rotation velocities and **(B)** transformed yaw and pitch rotation velocities.

Figure 4 shows a 10-second trace depicting rotation rates of the gaze-stabilization exercise for a pitch exercise. Figure 4A shows the rotation rates in the iPod’s frame of reference. Figure 4B shows the transformed rotation rates to earth-fixed frame of reference, using the formula shown in the “Validation of sensor measurements” section below. The transformed velocities confirm the primary movement in pitch.

### Patient interface

**Figure 5:**
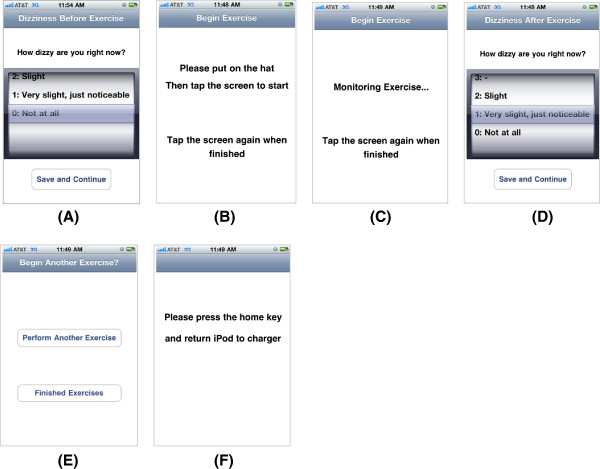
iPod interface screenshots; (A) User inputs initial dizziness severity rating, (B) User taps to start exercise, (C) Display during performance of exercise, (D) User inputs final dizziness severity rating, (E) User decides to continue or finish, (F) Reminder to exit application and charge iPod.

A series of simple displays on the iPod leads patients through each exercise (Figure 5A to F). When patients first turn on the iPod, a screen prompts them to enter their current severity of dizziness (A), using a picker wheel that is numbered from 0 to 10 with verbal descriptors. The picker wheel values were based on a numeric rating scale that is used in clinics. After they enter their pre-exercise dizziness, patients insert the iPod into the sleeve, put on the cap, and tap the screen anywhere to start (B). At the tap, a voice announces, “Begin”. They begin the exercise. During the exercise, the total duration of performance is announced every 10 seconds. When they finish, patients tap on the screen again and the voice announces, “Finished”. (C). They then take off the cap and enter their post-exercise dizziness rating (D). Patients are then asked whether they need to perform another repetition of the exercise (E). If they tap “Perform Another Exercise”, steps A through E are repeated. If they tap “Finished Exercises”, a screen reminds them to return the iPod to the charger (F).

For each exercise repetition, the data recorded by the application include the pre- and post-exercise dizziness severity ratings, duration of performance, timestamp (date and time) of when the exercise was performed, triaxial rotation velocity, and triaxial gravitational vector sampled at around 60 Hz.

### Validation of sensor measurements

Before conducting the usability study with patients, we validated the head-referenced yaw and pitch velocity measurements of the cap-based sensor system against a commercially available magnetic field motion-tracking system (Fastrak, Polhemus, Inc, Colchester, VT, RMS Static Accuracy 0.15 deg). Healthy control patients without a history of vestibular disease (six male, two female, ages 18–50) performed head movements that are similar to those used in vestibular rehabilitation. While performing the head movements, participants wore a plastic rock-climbing helmet to which the motion tracker and iPod were rigidly attached. To examine the effect of different orientations when the iPod was placed in the cap, the iPod could be oriented at one of three pitch inclinations from the horizontal (0, 45 and 90 degrees).

We asked each participant to perform head movements for 30 seconds under varying conditions: orientation of iPod (0, 45, 90 degrees), frequency of head motion (0.25, 0.5, 1 Hz), and direction of turning (pitch, yaw). The frequency of head turns was controlled by playing a metronome and asking the participants to move in synchrony with it. Each participant performed 18 trials to include all the combinations of the above, in randomized order. Each participant used one of three different iPods to test for consistency across iPods.

The motion tracker measured angular position in yaw, pitch and roll relative to an earth-fixed transmitter. The data were sampled at a fixed rate of 60 Hz using custom data acquisition software (National Instruments Labview). Data were lowpass filtered (phaseless 4^th^ order Butterworth filter, cutoff frequency = 4 Hz) and differentiated to compute rotation velocity using Matlab (Mathworks Inc).

The iPod provided the rotation velocity about its own X, Y and Z axes (Figure 3), using the rotationRate property of CMDeviceMotion in the iOS 4 SDK. We transformed the iPod-fixed rotation velocity into earth-fixed yaw and pitch rotation velocity by incorporating the gravity property of CMDeviceMotion:

$$\begin{array}{l} \text{yawVelocity}=-\left(\text{rotX}*\text{gravX}+\text{rotY}*\text{gravY}+\text{rotZ}*\text{gravZ}\right)\\ \text{pitchVelocity}=\sqrt{\text{rot}X^{2}+\text{rot}Y^{2}}*F\left(\text{rotX},\text{rotY}\right),\\ \text{where}:\\ F\left(\text{rotX},\text{rotY}\right)=\left\{\begin{array}{cc} \text{sign}\left(\text{rotY}\right) & \text{ifabs}\left(\text{rotY}\right)\geq \text{abs}(\text{rotX})\\ \text{sign}\left(\text{rotX}\right) & \text{otherwise} \end{array}\right\} \end{array}$$

where rotX stands for rotation velocity about the iPod X axis, and gravX stands for the component of the gravity vector along the iPod X axis.

The iPod does not sample data at fixed rates. However, a timestamp can be recorded when each sample is taken. Consequently, we transformed the iPod data to a fixed 60 Hz sampling rate using the cubic spline interpolation function in Matlab. In addition, the data were lowpass filtered (phaseless 4^th^ order Butterworth filter, cutoff frequency = 4 Hz).

For each trial, a correlation coefficient was computed to determine the strength of association between the magnetic field motion tracker and iPod measurements of yaw and pitch velocity, using the entire time series. The correlations between the measurements were high and consistent across all experimental conditions. Across all patients and trials, the mean correlation was 0.99 (standard deviation 0.005). Furthermore, the mean RMS error between the measurements was 3.4 deg/s (sd 5.5 deg/s), across a range of speeds from 58 to 178 deg/s. Therefore, we concluded that system measurements were valid.

### In-home patient study

To explore the usability of the iPod-based system to monitor gaze stabilization home exercise compliance and performance, we conducted a study of ten individuals with vestibular hypofunction who were receiving vestibular rehabilitation and performed gaze-stabilization exercises as part of their home exercise program. Table 1 describes patient demographics and clinical characteristics. Patients represented a typical population of people who have vestibular hypofunction. The age range was 28–67 years. Nine of the ten patients had unilateral loss, and one had bilateral vestibular loss. Most patients were receiving treatment within six months of their diagnosis, but one had chronic symptoms lasting 12 years. Patients had attended at least two sessions of vestibular rehabilitation before beginning the in-home trial, and thus had experience in performing the gaze-stabilization exercises in the clinic and at home. As can be seen in Table 1, the patients’ exercise prescriptions varied in exercise duration and daily frequency. The prescription for the number of repetitions for both yaw and pitch movements ranged from two to eight times per day, and the duration of exercise performance was either 30 or 60 s.

**Table 1:** Demographic and clinical characteristics of patients who participated in the in-home user trial

**ID**	**Sex**	**Age**	**Diagnosis**	**Duration of symptoms**	**DHI**	**Prescription**
1	F	59	Unilateral vestibular hypofunction	1 month	26	3×30 s
2	F	65	Right unilateral vestibular hypofunction, BPPV	12 years	42	2×30 s
3	F	67	Bilateral vestibular hypofunction	2 months	38	8×60 s
4	F	52	Right brain stem infarction	5 months	58	6×60 s
5	M	53	Left unilateral vestibular hypofunction	3 months	18	2×30 s
6	F	58	Left unilateral vestibular hypofunction	2 months	50	3×30 s
7	F	47	Right unilateral vestibular hypofunction	1 month	62	4×60 s
8	F	28	Right unilateral vestibular hypofunction, BPPV	1 month	58	6×60 s
9	M	54	Unilateral vestibular hypofunction	5 months	50	3×30 s
10	M	36	Right post-acoustic neuroma surgery	8 months	62	5×60 s

*DHI*, Dizziness Handicap Inventory [26].

*BPPV*, Benign Paroxysmal Positional Vertigo.

All participants were instructed in the use of the iPod device in the clinic by the first or second author after they had been given their exercise prescription on a printed sheet. Patients took home a small case containing the cap, iPod, charger, and printed instructions. They used the system for five to seven days. At the end of the seven-day trial, patients returned the device and completed an interview on its usability. They were asked how comfortable the cap was to wear, and whether it interfered with doing the exercises. They were also asked to write in any comments they might have or suggestions for improvement.

### Data analysis

The number of repetitions and duration of performance was tabulated and compared with each patient’s prescription. The mean dizziness ratings before and after each exercise were computed across the entire week. The velocity data were post-processed to determine the mean peak velocity in each direction for each trial, and then the mean and standard deviation of the peak values was calculated for each day and then over the entire week. Similarly, the descriptive statistics of the range of motion were obtained from the integral of the velocity data.

### Ethical approval

The experiment was conducted in accordance with the Declaration of Helsinki, and was carried out with the adequate understanding and written consent of the subjects. We certify that formal approval to conduct the experiments described has been obtained from the human subjects review board of the University of Pittsburgh (IRB #: PRO10100562) and Carnegie Mellon University (IRB #: HS11‒674).

## Results

Table 2 shows the compliance data from the in-home trial. Five of ten patients (patient IDs 1, 2, 3, 4, 9) were under-compliant on a majority of the days – they performed fewer exercises or for shorter durations than prescribed. Interestingly, the other five patients were generally above-compliant. Even though patient #8 did not perform exercises in the pitch plane, he did many more yaw exercises than prescribed; he also had the highest self-reported symptom levels, as shown in Table 3.

**Table 2:** Compliance data of patients in the in-home user study

**Patient**	**Prescription**	**Direction**	**Day 1**	**Day 2**	**Day 3**	**Day 4**	**Day 5**	**Day 6**	**Day 7**
**1**	3×30 s	**Yaw**	1×60	1×60	1×60	1×60	1×60	1×60	Stopped*
		**Pitch**	0	1×60	1×60	1×60	1×60	1×60	
**2**	2×30 s	**Yaw**	1×30	1×30	3×30	2×30	1×30	1×30	3×30
		**Pitch**	1×30	1×30	3×30	2×30	1×30	1×30	3×30
**3**	8×60 s	**Yaw**	1×60	1×60	1×60	2×60	2×60	1×60	2×60
		**Pitch**	1×60	1×60	1×60	2×60	2×60	1×60	2×60
**4**	6×60 s	**Yaw**	1×30	1×50	1×30	2×30	1×30	1×30	Returned early
		**Pitch**	0	1×50	1×30	0	1×30	1×30	
**5**	2×30 s	**Yaw**	2×60	4×60	4×60	4×60	4×60	4×60	4×60
		**Pitch**	2×60	4×60	4×60	4×60	4×60	4×60	4×60
**6**	3×30 s	**Yaw**	3×30	6×30	3×30	3×30	5×30	6×30	3×30
		**Pitch**	1×30	5×30	4×30	3×30	0	6×30	3×30
**7**	4×60 s	**Yaw**	5×60	4×60	4×90	4×90	4×120	Returned early	
		**Pitch**	4×60	4×60	4×90	4×90	4×120		
**8**	6×60 s	**Yaw**	9×60	18×60	25×60	13×60	iPod error**		
		**Pitch**	0	0	0	0			
**9**	3×30 s	**Yaw**	2×30	3×30	3×30	1×30	2×30	2×30	1×30
		**Pitch**	0	3×30	3×30	1×30	2×30	2×30	1×30
**10**	5×60 s	**Yaw**	4×60	6×60	10×60	5×60	5×60	iPod error**	
		**Pitch**	3×60	7×60	10×60	5×60	7×60		

*Patient stopped using the device for fear of acquiring brain cancer from iPod device.

**iPod contained a software bug which caused a crash when saving data; data from these sessions were lost.

**Table 3:** Dizziness severity rating (out of 10) before and after each exercise for each patient, averaged over all trials and days of exercise performance

	**Yaw**			**Pitch**		
**Subj**	**Pre**	**Post**	**Change**	**Pre**	**Post**	**Change**
1	0.2	1.8	1.6	0.6	1.6	1.0
2	0.8	1.5	0.7	0.9	1.2	0.3
3	2.7	3.6	0.9	2.9	3.0	0.1
4	1.3	1.8	0.5	2.0	2.3	0.3
5	1.3	1.9	0.6	1.6	1.9	0.3
6	1.9	2	0.1	2.6	2.7	0.1
7	2.5	3.1	0.7	3.0	3.7	0.7
8	4.7	5.1	0.4	N/A*	N/A*	N/A*
9	3.0	2.3	−0.7	2.5	2.1	−0.4
10	1.1	2.5	1.4	1.1	1.2	0.1
Group	2	2.6	0.6	1.9	2.2	0.3

*This patient did not do pitch exercises.

**Figure 6:**
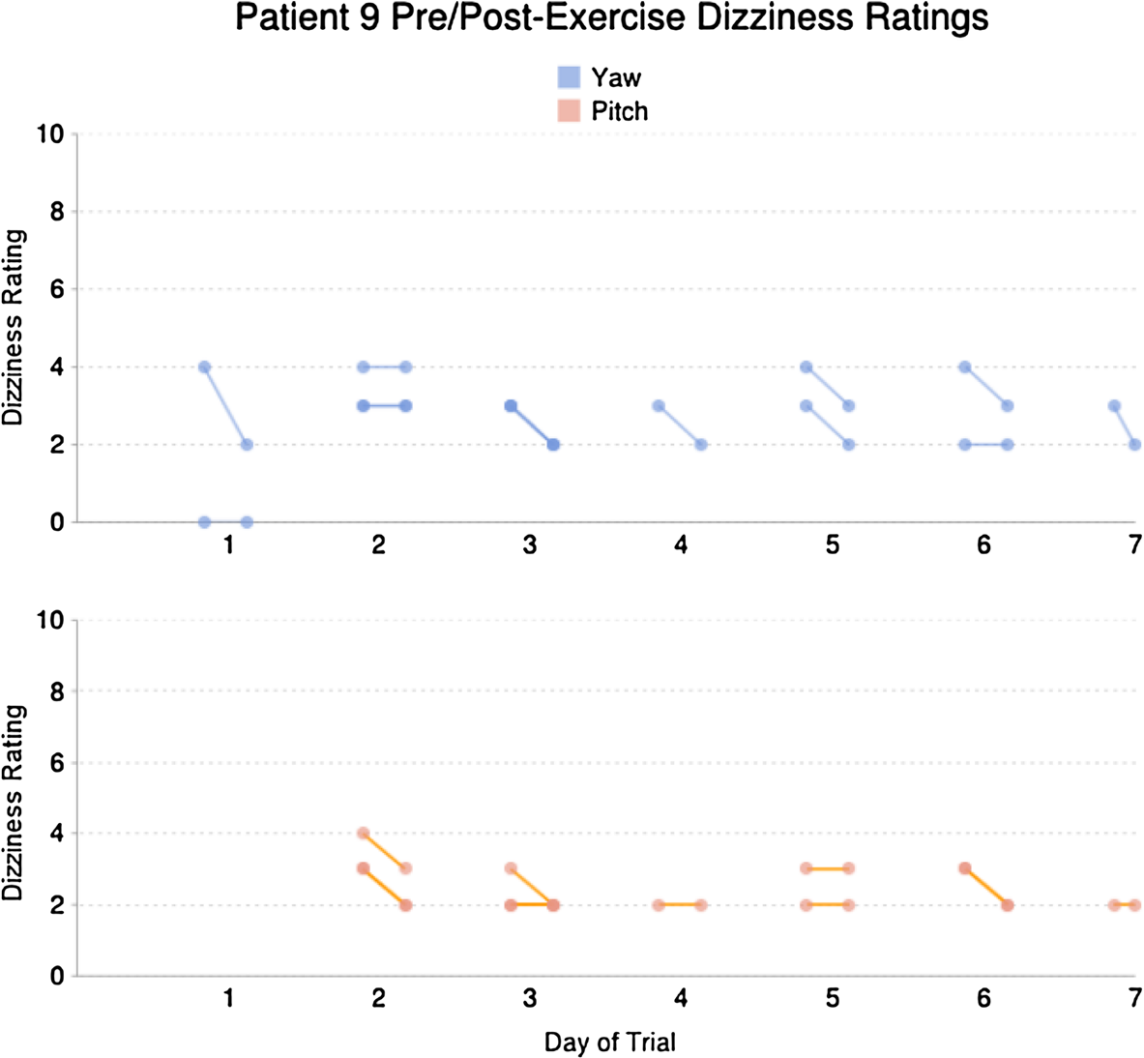
**Example display of dizziness severity as a function of day of exercise performance; for each pair of connected dots, the left dot represents the pre-exercise rating and the right dot represents the post-exercise rating.** When exercises on the same day have the same pre- or post-exercise ratings, the dots overlap and become darker.

Patients started off with a low mean dizziness severity rating prior to performing the gaze-stabilization exercises, about level 2 (equated with “slight” dizziness, Table 3). The lowest and highest values were 0.2 and 4.7. Immediately after the exercise was performed, yaw movements induced slightly more dizziness than pitch movements (increase of 0.6 points v. 0.3 points) on average. Using the iPod-based system, we could track daily dizziness ratings, as shown in Figure 6. Here it can be seen that patient #9’s dizziness either stayed the same or decreased after each exercise.

**Figure 7:**
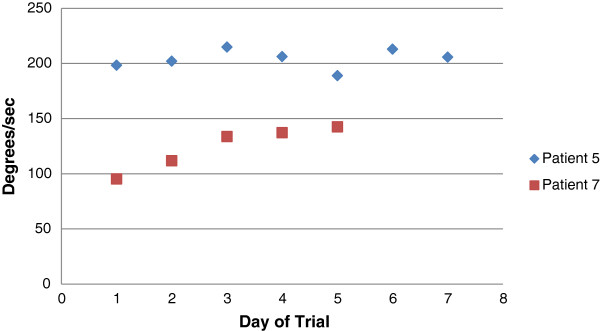
Average peak yaw head velocity in two patients for each day of the study.

The peak velocity and range of motion in the yaw and pitch planes are detailed in Tables 4 and 5. The data indicate considerable inter-subject variability in the mean values of the head velocity (98 to 204 deg/s in the yaw plane), as well as substantial intra-subject variability in day-to-day head velocity performance (e.g. a range of 113 to 222 deg/s for patient #3). In the pitch plane, the average velocity of head movement was lower. The data can be used to examine day-to-day trends in velocity of movement (Figure 7). For example, patient #5 consistently moved at around 200 deg/s, whereas patient #7 increased her velocity throughout the week from 95 to 142 deg/s. Range of motion in the yaw and pitch planes demonstrated similar inter- and intra-patient variability (Table 5).

**Table 4:** Peak velocity (degrees per second) for each patient, averaged over all trials and days of exercise performance

	**Yaw**			**Pitch**		
**Patient**	**Mean**	**SD**	**Range**	**Mean**	**SD**	**Range**
1	105	19	71 - 127	84	4	77 - 89
2	141	22	105 - 176	93	17	57 - 116
3	168	31	113 - 222	91	23	66 - 121
4	158	10	144 - 170	114	16	90 - 129
5	204	8	188 - 214	157	6	147 - 166
6	98	13	73 - 117	53	13	28 - 65
7	124	18	95 - 142	113	13	90 - 124
8	121	14	106 - 142	N/A*	N/A*	N/A*
9	100	17	83 - 147	78	5	68 - 85
10	183	38	122 - 249	129	32	76 - 168
**Group**	**140**	**35**		**101**	**29**	

*This patient did not do pitch exercises.

**Table 5:** Range of motion (degrees) for each patient, averaged over all trials and days of exercise performance

	**Yaw**			**Pitch**		
**Patient**	**Mean**	**SD**	**Range**	**Mean**	**SD**	**Range**
1	72	15	49 - 101	63	6	56 - 71
2	66	9	44 - 74	31	7	16 - 36
3	49	13	31 - 75	23	12	14 - 49
4	78	22	56 - 124	38	3	34 - 41
5	61	2	57 - 64	52	1	50 - 53
6	24	4	20 - 32	9	2	5 – 11
7	60	6	50 - 67	63	4	56 - 66
8	47	4	41 - 50	N/A*	N/A*	N/A*
9	34	3	29 - 41	24	2	20 - 26
10	64	9	45 - 75	56	3	51 - 60
**Group**	56	16		40	18	

*This patient did not do pitch exercises.

### Usability results

At the end of their in-home trial, the patients were given a questionnaire to assess the acceptability and usability of the cap and device. The results suggest that the system is feasible for in-home use.

In response to the question, “How comfortable was the cap to wear?” two patients reported “Very Comfortable”, six patients reported “Comfortable”, and two patients reported “Neutral”. No patients selected “Uncomfortable” or “Very Uncomfortable”. To the question, “Did the hat interfere with your exercises?” five patients reported “Not at all”. The other five patients reported “A little”. No one selected “A lot”. When interviewed, those who reported “A little” said that the interference was caused by the cap being too loose; it sometimes drooped and prompted them to adjust it. We chose a universal-adjustable cap with a Velcro fastener and assumed that it would fit all patients. In the future, caps of various sizes will be provided. One patient felt that the weight of the iPod was too heavy, suggesting that it contributed to the hat shifting slightly when he performed the pitch exercises.

### PT feedback

The PT Dashboard visualizations were presented on the iPad to four PTs who were not involved in the creation of the system. They were shown hypothetical patient data, as would be gathered with the system. We used hypothetical data in order to intentionally insert problematic performances to see if the visualizations were effective in communicating these problems. The PTs were asked to “think aloud” as they reviewed each hypothetical patient chart, including those for duration, dizziness rating, velocity, range of motion and head-turn frequency. All four PTs were able to find the problems regarding skipping sessions (shown in the Duration chart), small range of motion, and unchanging dizziness symptom ratings perhaps indicating that the prescription parameters were not challenging enough. Overall, they found the visualizations easy to understand. One PT suggested that the velocity values be changed to “slow, medium, fast” because she found values such as “65 degrees/s” to be difficult to interpret. Another PT, however, found the numerical values helpful. Another suggestion among PTs was a summary screen to see all of the metrics at a glance. This screen could highlight problems that PTs should explore further. When asked about the value of the system, PTs suggested that the patient data might increase accountability for the patient, increase the PT’s understanding of the patient, and assist in patient documentation and patient sharing (where multiple PTs treat the same patient).

## Discussion

In this paper, we have reported the development and validation of an iPod-based application for monitoring the compliance and performance of gaze-stabilization exercises in a sample of individuals with vestibular disorders. User tests showed that the device was easy to use and comfortable to wear. Quantitative analysis showed that useful metrics can be extracted from the registered motion data.

### Validation

The iPod sensors were validated against a gold standard (Polhemus magnetic field-tracking system) for accuracy. The average correlation was above 0.99 for the 142 validation trials, showing that the iPod can be used to document head velocity for this application.

### Compliance data

Compliance results showed data that might not be captured in patients’ own retrospective self-reports, e.g., that five of the patients exercised more frequently than prescribed. (One patient exercised significantly more, peaking at 25 sets of horizontal exercises in one day compared to the prescribed six sets). General physical therapy exercise compliance has been explored in previous studies. For example, Sluijs et al. surveyed 300 PTs in various domains about their patients’ compliance rates; these rates were measured by patient retrospective self-report [27]. The study found that non-compliance rates might be as high as 70%. However, objective quantitative compliance data, such as the data presented in this paper, have not been documented for this population. The over-compliance phenomenon, especially spikes such as can be seen in Patient #8’s third day when he performed 25 sets compared to the prescribed 6, has not been documented. This gulf in measurement is perhaps due to the nature of self-report questionnaires, which commonly ask if patients have done their exercises regularly or not. Objective quantification shows exact daily frequencies and can more accurately report both expected and unexpected phenomena.

### Performance data

As noted above, head movement metrics (range and velocity) documented substantial variability, both inter-subject and intra-subject. Inter-subject variability was evidenced by mean values of head velocity ranging from 98 to 204 deg/s in the yaw plane. Intra-subject variability was exemplified by patient #3, who showed a head velocity range of 113 to 222 deg/s throughout the trial. Similar variability was shown among the patients for range of motion, ranging from 20 to 124 degrees for yaw direction and 5 to 71 degrees for pitch direction (detailed in Table 5). Documenting such variation may be of considerable importance in customizing standard exercises to individual patient needs and responses to treatment prescriptions.

### Dizziness ratings

Another metric recorded by the device was the patient’s dizziness rating before and after each exercise. These daily ratings are important to PTs because they show the effects of exercise on a more detailed level. Physical therapists conventionally ask patients to record these ratings in paper diaries; PTs interviewed reported that this approach has a very low compliance rate, although exact numbers have not been documented. However, researchers have documented paper-diary compliance rates of other populations, such as pain patients. In a study by Stone et al., it was shown that paper diaries had only an 11% compliance rate [28]. In addition, the study showed that an electronic diary, such as the dizziness-rating logging function in our system, which can time-stamp entries automatically, yielded a much higher compliance rate (94%). The study suggests that automatic time-stamping prevents fake diary construction and motivates patients through accountability.

### Usability

The self-contained design of the device – an iPod inserted into a sleeve on a cap – minimized setup complexity and promoted its ease of use. The user interface flow was also designed to minimize complexity, and voice output was provided to guide patients through the exercises. Patients largely reported that the device was easy to use, and no patients needed technical support during the trial.

To the open-ended question, “Please give any suggestions for improvement”, seven patients stated they had none. One patient stated, “It was very easy to use. I am technically challenged and I had no problem with it.” Two patients suggested adding more auditory feedback to guide the exercises, such as beeps that confirm proper head turns.

### Motivation

Physical therapists who reviewed the Dashboard stated that the system could increase patients’ motivation by showing them incremental progress they could not see before. In addition, the system could support collaborative goal-setting between PTs and patients. Goal-setting and information visualization has been used to motivate behavioral change in other domains, such as sustainability [29]. PTs also stated that accountability could improve motivation as well. This view was shared by a patient who said during the interview, “It was more motivating to do the exercise knowing that I was accountable… that it was going to record whether I did it or not. People should do it for all exercises; then they wouldn't skip so much.”

### Clinical relevance

The motivating factor for developing this application was to optimize the prescription of gaze-stabilization exercises so that individuals with vestibular dysfunction could progress and recover more quickly. Several important features of the application could facilitate this process.

First, having a record of the duration and number of exercise repetitions, and being able to correlate this information with dizziness severity ratings, will allow the physical therapist and patient to discuss this information and decide on the best treatment plan going forward. Referring back to Figure 6, we can surmise that patient #9 was tolerating the gaze-stabilization exercises. Upon seeing this information, the therapist would probably progress the prescription to increase the velocity or the number of repetitions. Furthermore, the therapist could inquire about other circumstances that might explain the increased symptoms on those days. This recorded information represents an improvement over patient recall, which is often inaccurate [30]. While the same information could be entered into an exercise diary, using the iPod device may relieve the patient of the burden of remembering to log the information.

Another benefit is that the velocity of head movement has heretofore been largely ignored as a part of the prescription process, primarily because there has been no easy way to measure it at home. It is important to note that in this study, velocity of head movement was not prescribed by the physical therapist. Rather, the physical therapist usually asked the patients to perform the exercise at a comfortable speed. The function of the vestibulo-ocular reflex is to stabilize images on the retina at velocities of up to 350 deg/s [31], and frequencies up to 5 Hz [32]. It is therefore important for people with vestibular disease to perform exercises at a variety of speeds and frequencies, so that they recover their full range of function. The system can provide this critical information, and future versions may incorporate real-time feedback so that users know the velocity at which they are moving their heads with each repetition. Using this system, physical therapists and patients would be able to view and correlate dizziness severity with head velocity, and adjust head velocity accordingly. Therapists could also examine the data to determine whether users were performing an exercise incorrectly by checking for any out-of-plane movements, e.g. tilting the head side-to-side.

### Limitations

Although the system we devised can track head movements, it cannot determine if patients are keeping their eyes fixated on a target, as they are directed to do. Usually with a short duration of in-clinic instruction of the gaze stabilization exercise, patients are able to maintain gaze fixation on the target. A limitation of the in-home study is that the participant sample size of 10 is small and may not be fully representative of the people who would be prescribed the exercises. The time period of seven days is shorter than typically necessary for a full evaluation; our goal for the user study was to inform design and assess feasibility.

### Conclusion and future work

In this paper, we demonstrated the potential for a mobile consumer device, the iPod Touch 4G, to be used to measure home gaze-stabilization exercise compliance. We presented a sensor-based mobile system consisting of an iPod fitted in a baseball cap to be worn during the exercise. The system was designed to monitor and extract relevant metrics for assessing compliance, performance, and symptom levels. We validated the sensors’ accuracy against a gold standard, and conducted a user study to assess the device’s in-situ feasibility. The validation study showed that the iPod sensors can be used to monitor the exercises with high accuracy and repeatability. The in-home user study showed that the device is easy to use and comfortable to wear in a population that includes elderly patients. Quantitative analysis showed that the necessary exercise metrics can be extracted from the performance data. Physical therapists believed that use of the system could improve patient motivations for performing the exercises.

In the future, we plan to continue developing the system and include real-time coaching. Having the sensing infrastructure in place allows for not only passive measuring and reporting of exercise performance but active intervention as well. We plan to work with physical therapists to develop customizable performance standards for each patient to target.

## Competing interests

The authors declare that they have no competing interests.

## Authors’ contributions

KH conceived of the iPod-based system. KH and PS drafted the research design, which was refined in collaboration with SK, DS and AS. KH and PS carried out data collection and data analyses. All authors assisted in the interpretation of results. KH and PS drafted the manuscript, which was edited by SK, DS and AS. All authors participated in revisions, and read and approved the final manuscript.
